# Chip-integrated optical power limiter based on an all-passive micro-ring resonator

**DOI:** 10.1038/srep06676

**Published:** 2014-10-20

**Authors:** Siqi Yan, Jianji Dong, Aoling Zheng, Xinliang Zhang

**Affiliations:** 1 Wuhan National Laboratory for Optoelectronics, Huazhong University of Science and Technology, Wuhan, China, 430074

## Abstract

Recent progress in silicon nanophotonics has dramatically advanced the possible realization of large-scale on-chip optical interconnects integration. Adopting photons as information carriers can break the performance bottleneck of electronic integrated circuit such as serious thermal losses and poor process rates. However, in integrated photonics circuits, few reported work can impose an upper limit of optical power therefore prevent the optical device from harm caused by high power. In this study, we experimentally demonstrate a feasible integrated scheme based on a single all-passive micro-ring resonator to realize the optical power limitation which has a similar function of current limiting circuit in electronics. Besides, we analyze the performance of optical power limiter at various signal bit rates. The results show that the proposed device can limit the signal power effectively at a bit rate up to 20 Gbit/s without deteriorating the signal. Meanwhile, this ultra-compact silicon device can be completely compatible with the electronic technology (typically complementary metal-oxide semiconductor technology), which may pave the way of very large scale integrated photonic circuits for all-optical information processors and artificial intelligence systems.

Due to the ultrahigh-speed and ultrawide-band brought by adopting photons as information carriers, photonic integration has been a long-term pursuit for researchers, which can break the performance bottleneck^1^,^2^ incurred in modern semiconductor-based electronic integrated circuit (IC) such as thermal losses and poor process rates^3^,^4^. In recent years, many optical integration devices have been demonstrated as counterparts of the basic “functionalities” in electronic circuit, including all-optical logic gates^5^,^6^,^7^, optical differentiation^8^,^9^,^10^,^11^,^12^, optical integration^13^,^14^,^15^,^16^, optical random access memory (RAM)^17^,^18^, optical logic discriminator^19^, optical comparator^20^, as well as all-optical ordinary differential equation (ODE) solvers^21^,^22^,^23^. The dramatic advances in integrated optics open up the possibilities to replace the traditional electronic IC with photonic IC. It is worth noting that current limiting circuit^24^ is widely used as protection of the load against the harmful effects due to short-circuit or similar problem by imposing an upper limit of the current in the load. Meanwhile, most optical devices are very vulnerable to high optical power. Unfortunately, few reported work was focused on the counterpart of current limiting circuit to protect the optical device from harmful effect caused by high power. Recently, Makri theoretically proposed the concept of reflective power limiter based on nonlinear localized modes^25^, where a nonlinear layer was sandwiched by two reflective mirrors, thus increased the device complexity, and no experiment was carried out to verify the performances of the power limiter.

In this study, we propose and experimentally demonstrate an all-passive chip-integrated optical power limiter based on a single silicon micro-ring resonator (MRR). Figure 1(a) gives a general view about the proposed optical power limiter. The power limiter is composed of one ring waveguide and two straight waveguides, together forming an add-drop MRR. The essential principle is a nonlinear power attenuation induced by MRR red shift due to thermo-optical effects. For the first time, Carmon et al. established the well-known theoretical model of optically induced thermal effects in high-Q micro-cavity community^26^. The theoretical model indicated that the thermal effects will cause a red-shift of the resonance and the red-shift response can attain a steady state finally. The equilibrium between the absorbed- and dissipated-heat of the micro-cavity is described as follows 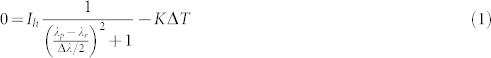

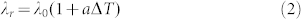
where *I_h_* is the optical power that heats the cavity, *λ_p_*, *λ*_0_ and *λ_r_* represent the input wavelength, the cold cavity resonance-wavelength and resonance wavelength after red-shift respectively, *a* is the temperature coefficient of resonance-wavelength, Δ*T* is the temperature difference between mode volume and the surrounding, *K* (J/s°C) is the thermal conductivity between the cavity mode volume and the surrounding. For a given pump light with the wavelength fixed at the cold cavity resonance-wavelength, one stable solution of Δ*T* for Eq. (1) can be calculated, inducing a stable red-shift of the resonance described as Eq. (2).

Figure 1(b) calculated the red–shift and the transmittance of silicon MRR as a function of input power according to Eq. (1) and (2). In the calculation, we assume that *λ_p_* = *λ*_0_, the quality factor (Q) of the MRR is 15000 and drop port transmittance has a −10 dB loss at the resonant wavelength considering coupling losses. For silicon materials, the temperature coefficient of resonance wavelength is 5.34*10-5 (1/°C) and K is 6.32(J/s°C). As shown in Fig. 1(b), when the input power is low, the red shift is too small to affect the transmittance significantly. On the contrary, when the input power is high enough to cause a large red-shift, the transmittance starts to decrease remarkably. We define the input power where the MRR transmittance reduces to −15 dB as threshold power. The relationship between the output power and input power is also calculated, as depicted in Fig. 1(c). The result indicates that beyond the defined threshold power the output power stops increasing with the input power and even starts to decrease conversely. Thus the optical power limiter imposes an upper limit on the output power through the MRR. Although most optical amplifiers including erbium-doped optical fiber amplifiers (EDFAs) have a maximum output power (namely saturated power) due to the gain saturation^27^, they are active elements and provide gain for the input signal, which means they cannot prevent the optical device from damaging with high power. Oppositely, they are prone to damage the optical device because the maximum power of optical amplifiers is often very high. Besides, they can hardly be integrated with silicon-on-insulator (SOI) chip. However, the proposed optical power limiter here is all-passive, chip-scale and can protect the optical device from high power damaging effectively without impairing the input signal.

## Results

### Device structure

**Figure 2:**
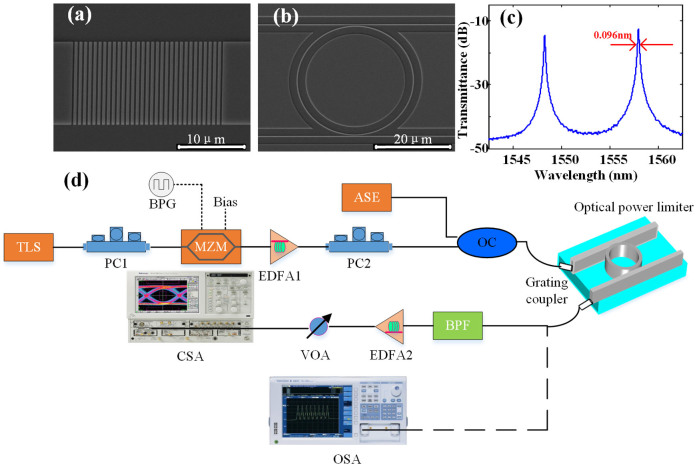
MRR design and experimental setup. (a) Microscope image of the grating coupler, (b) microscope image of the fabricated MRR, (c) measured transmission spectrum of the MRR (d) schematic diagram of the experimental setup.

The optical power limiter, consisting of a ring waveguide and two straight waveguides, is fabricated on the commercial SOI wafer. The optical signal is coupled into the MRR by vertical grating couplers. Figures 2(a) and 2(b) show the scanning electron microscope (SEM) images of the grating coupler, and the fabricated MRR, respectively. To fabricate the device, the upper 340 nm silicon layer was etched downward for 240 nm to form a silicon ridge waveguide and input/output grating couplers, through inductively coupled plasma etching. The coupling loss of the grating coupler was measured to be 5 dB for a single side. The grating coupler has a period of 630 nm and the duty cycle is 56%. The radius and the width of the ring are about 12 μm and 525 nm, respectively. The coupling gap between the bus waveguide and bending waveguide of MRR is about 330 nm. Finally, we measure the Q factor of the fabricated MRR, which is about 18000, as shown in Fig. 2(c).

### Experimental overview

To study the performance of optical power limiter, we first measured the output power as a function of input power and the transmission spectra at the drop port for both continuous wave (CW) injection, 10 Gbit/s and 20 Gbit/s non-return-to-zero on-off-keying (NRZ-OOK) modulated signal successively. Meanwhile, the time domain measurement of modulated output signal is performed to figure out whether the optical power limiter affects the signal quality. The experiment setup is shown in Fig. 2(d). In the experiment, a CW light is emitted by a tunable laser source (TLS) with a tuning resolution of 0.01 nm, which enables us to precisely align to the resonance wavelength of the MRR. We first measure with CW light injection. Then the CW light is modulated by cascaded Mach-Zehnder modulators (MZMs) driven with self-coded data signal from a bit pattern generator (BPG) as input signal. We adjust the MZMs and BPG to generate 10 Gbit/s and 20 Gbit/s NRZ-OOK signal respectively. Subsequently, the data signal is amplified by a high-power EDFA. To measure the resonance shift of the MRR, an amplified spontaneous emission (ASE) source is coupled with the input signal by an optical coupler (OC) before light is coupled into the MRR with vertical grating coupler. The ASE source is used to monitor the spectral shift of MRR transmission. The output power is measured by recording the peak power of the transmission spectrum at input wavelength. The optical spectrum analyzer (OSA, YOKOGAWA AQ6370C) can record signal with a maximum power of 25 dBm, which ensures that the output power is correct without saturation. Meanwhile, the temporal signal is filtered out by a band pass filter (BPF) and amplified by another EDFA before it is analyzed by a communication signal analyzer (Tektronix CSA 8000B) with an optical bandwidth of 40 GHz.

### Experimental results

**Figure 3:**
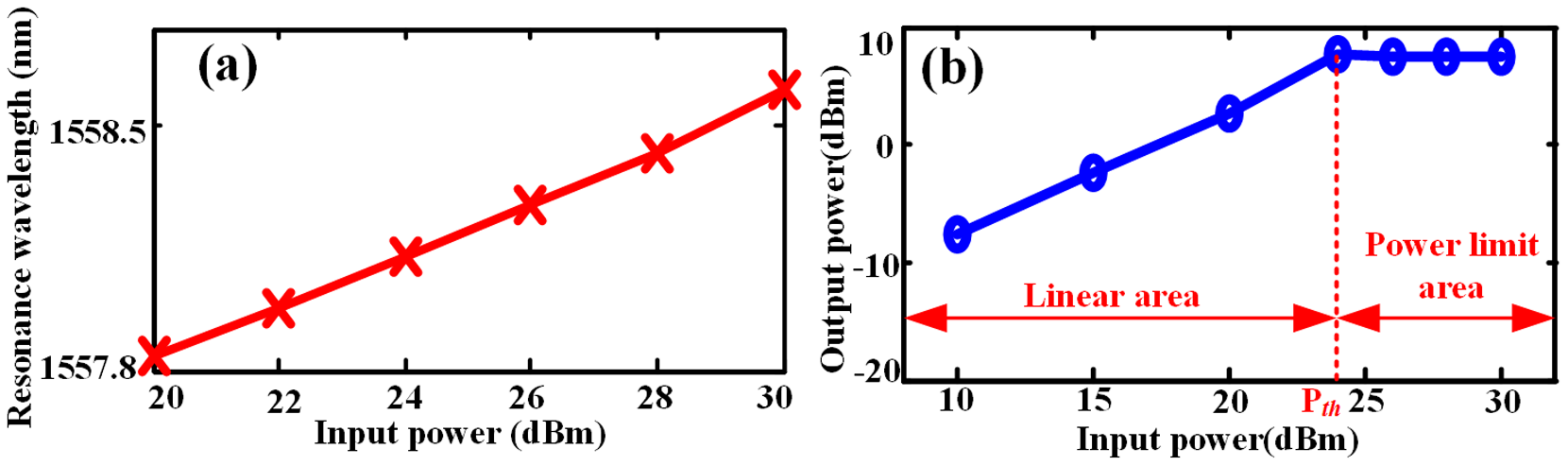
Experimental results for CW signal. (a) The resonance wavelength as function of input power, (b) the output peak power as function of input power.

We first measure the performance of optical power limiter under CW input. Figure 3(a) depicts the measured resonant wavelength with continuous wave (CW) light input under various power injections. It is clearly shown that the resonant wavelength experiences a red-shift due to the thermal effect. When no input signal is applied on the MRR, the 3-dB bandwidth is around 0.096 nm with a resonance wavelength of 1557.87 nm, while the applied power is increased to 30 dBm, the 3-dB bandwidth remains unchanged with the resonant wavelength shifting to 1558.568 nm. Figure 3(b) illustrates the relationship between input power and output power. When the input power is lower than the threshold power, the red-shift is relatively small. Therefore, the output power increases linearly with the input power, where we define as linear area. The output power stops increasing and nearly remains unchanged when the input power is higher than threshold power due to the large red shift of the resonance peak. Thus the output power is effectively restrained and we define the area where the power is higher than the threshold power as power limit area. Thanks to the existence of the power limit area, the following optical devices of optical power limiter can be effectively protected from high power damaging.

**Figure 4:**
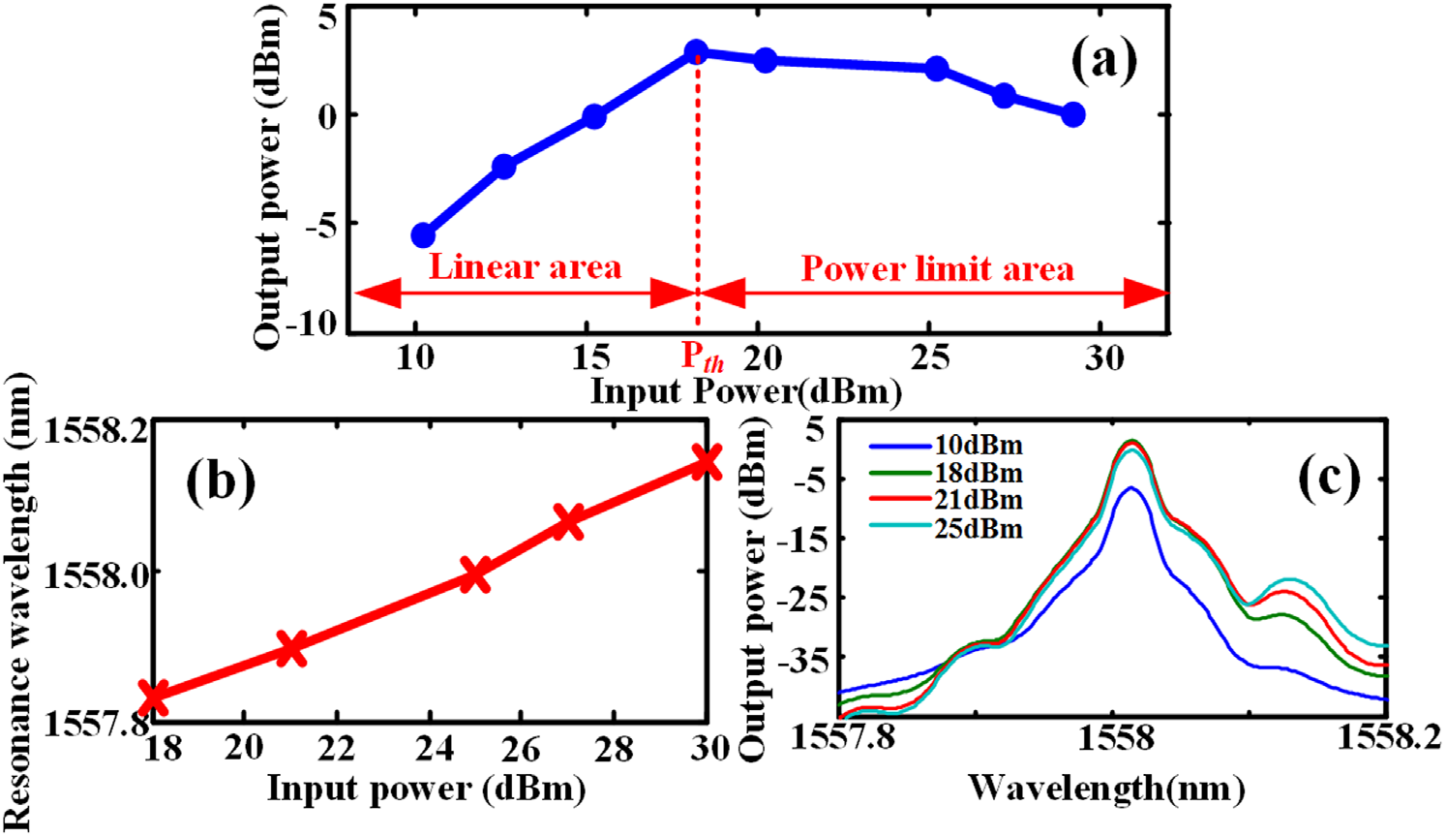
Experimental results for 10G bit/s signal. (a) The output peak power as function of input power, (b) the resonance wavelength as function of input power, (c) measured transmission spectra around 1558 nm, indicating the signal wavelength.

Next, we employ the NRZ-OOK modulated signal as input. When the signal bit rate is set at 10 Gbit/s with the wavelength of 1557.87 nm, the measured output power as a function of input power is shown in Fig. 4(a). We employ the input power as 10 dBm, 12 dBm, 15.2 dBm, 18 dBm, 21 dBm, 25 dBm, 27 dBm, and 30 dBm, respectively. As can be seen in Fig. 4(a), the output power of optical power limiter stops increasing when the input power is higher than 18 dBm. Therefore, the signal through the optical power limiter can have an output power of no more than 3 dBm even if the input power is as much as 30 dBm, which reveals that the optical power limiter can impose an upper limit for input signal with arbitrary power. To clearly demonstrate that this power limiting phenomenon is caused by the red-shift, we measure the resonant wavelength as a function of input power, as shown in Fig. 4(b). We can find out that the whole transmission spectrum moves to longer wavelength as the input power increases, thus verifying that it is the red-shift that leads to the power limiting phenomenon. However, we noticed that the red shift here is about 0.25 nm when the input power is 25 dBm, which is smaller than the case of CW input. Meanwhile, the transmission spectrum around signal wavelength is also measured, as shown in Fig. 4(c). One can see that when the input power is as high as 25 dBm, the output peak power is a little bit lower than that of 21 dBm input. This further proves that optical power limiter can effectively limit the output power thus protect optical device from high power damage.

**Figure 5:**
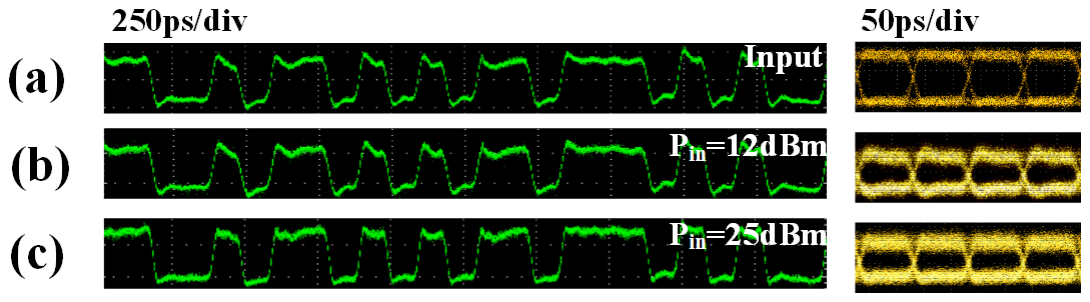
Time domain measurement of the signal. (a) The input signal and eye-diagram, (b) the output signal when the input power is 12 dBm, (c) the output signal when the input power is 25 dBm.

The measured temporal waveforms are illustrated in Fig. 5. Figure 5(a) shows the input waveform and eye diagram with pseudo-random binary sequence (PRBS) at a bit rate of 10 Gbit/s. When the input power is 12 dBm, which is in linear area, the output waveform is shown in Fig. 5(b). The measured signal to noise ratio (SNR) of output signal here is 61 dB. When the input power is set in power limit area, the measured waveform is displayed in Fig. 5(c). The measured SNR of output signal in power limit area is 63 dB. The small difference of the SNR may result from the high-power EDFA, which is capable of boosting the SNR of on-off signal. Thus, one can see from both SNR and eye diagram that no matter the input power is in linear area or power limit area, the output signal is almost free from deterioration.

**Figure 6:**
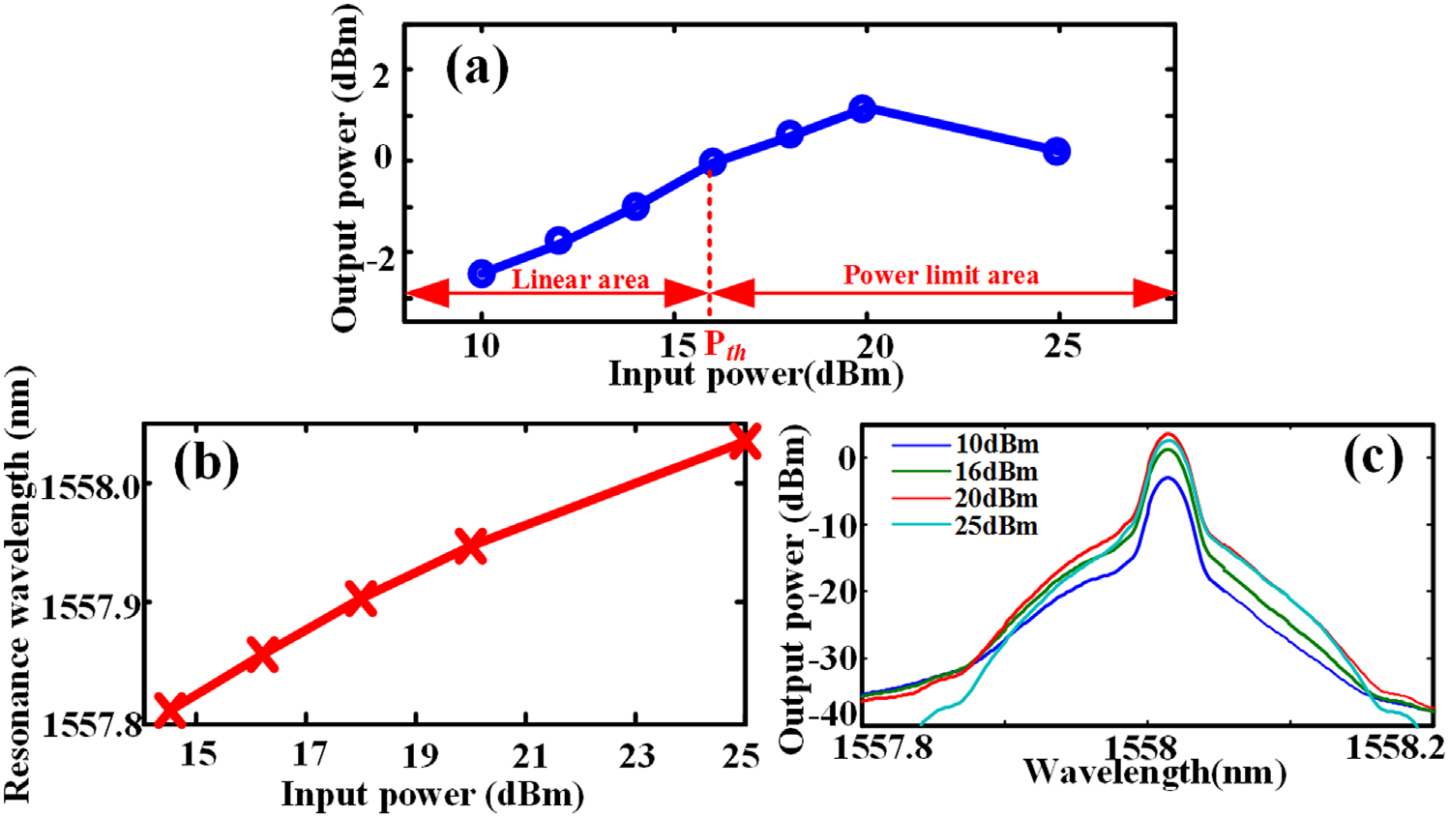
Experimental results for 20 Gbit/s signal. (a) The output peak power as function of input power, (b) the resonance wavelength as function of input power, (c) measured transmission spectra around 1558 nm, indicating the signal wavelength.

Next we increase the signal bit rate to 20 Gbit/s. The measured output power against input power is depicted in Fig. 6(a). The input power is set at 10 dBm, 12.4 dBm, 14.5 dBm, 16.2 dBm, 18 dBm, 20 dBm, and 25 dBm, respectively. The measured threshold power in this case is about 16 dBm. Despite that the output power actually does not reach the maximum until the input power is 20 dBm, the output power is linearly related to the input power till the input power increases to 16 dBm, which means the transmittance decreases to -15 dBm when the input power is 16 dBm. Similar to the case of 10 Gbit/s, the upper limit of output power is 6 dBm. Then, we measure the resonance wavelength as a function of input power, as shown in Fig. 6(b). One can see the red-shift turns to be smaller than the case of Fig. 4(b). Figure 6(c) shows the output signal spectra at different launched power. The output power reaches the maximum at 20 dBm launched power and starts to decrease afterwards. Therefore, the power limiter is still working when the input signal bit rate is 20 Gbit/s.

**Figure 7:**
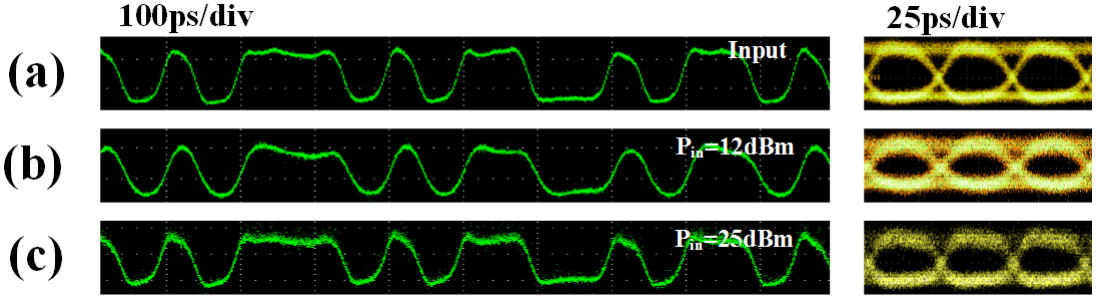
Time domain measurement of the signal. (a) The input signal and eye-diagram, (b) the output signal when the input power is 12 dBm, (c) the output signal when the input power is 25 dBm.

Figure 7 shows the temporal waveforms of output signals. The measured SNR for output in linear area and power limit area are 55 dB and 58 dB, respectively. It can be seen that the output signal is still well maintained in both linear area and power limit area.

## Discussion

**Figure 8:**
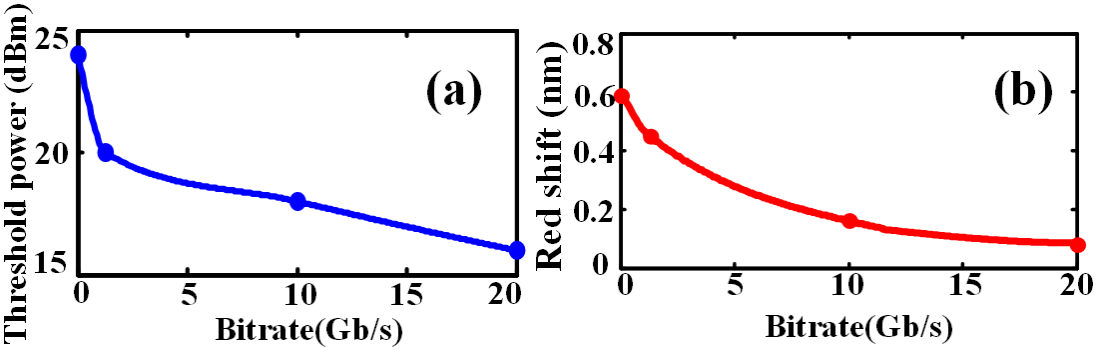
Effect of signal rates on performance of the optical power limiter. (a) The effect on threshold power, (b) the effect on amount of the red shift.

Based on the measurements above, we find that some important characteristics of the optical power limiter including the threshold power and the amount of red-shift are related to input signal bit rate. To thoroughly investigate the relationship between the input signal bit rate and the optical power limiter performance, we first measure the threshold power under different bit rate as shown in Fig. 8(a). One can see that the threshold power decreases along with the input signal bit rate increasing. We believe that the bandwidth of MRR and input signal are jointly responsible for this phenomenon. The 3dB-bandwidth of the fabricated MRR is rather narrow. Meanwhile, input signal with higher bit rate possesses higher bandwidth, which may exceed the bandwidth range of the MRR. Thus for the case of signal with higher bandwidth, more energy may be attenuated by the MRR, making an additional nonlinear attenuation for the output power relevant to the input power.

We also measured the amount of red-shift under different bit rates, as shown in Fig. 8(b). The input optical power is fixed at 25 dBm. One can see that higher speed signal can generate less red-shift. The reason is that thermal effect is a cumulative process, higher speed signal means the MRR can experience shorter heating process than lower speed signal thus the refractive change is lower. Therefore the red-shift of higher bit rate signal is not as much as lower bit rate input. Apparently, CW input light can generate the largest red-shift because heating process is continuous, as Fig. 8(b) has verified. Despite that higher speed signal generates less red-shift, the optical power limiter is still capable of limiting power of ultrahigh speed signal because excessively high power input signal can also generate significantly red-shift to restrain output power.

Another important parameter of the optical power limiter is the Q factor. The operation bandwidth is actually restrained by MRR 3 dB bandwidth, which is 0.086 nm. In the measured results above, we have demonstrated that the device is capable of limiting the output power at 20 Gbit/s effectively. This may be the highest bit rate signal for the fabricated device to process. To break through the speed limitation of the optical power limiter, one can design an add-drop MRR resonator with a lower Q factor. However, the red-shift may be reduced as well because of the weaker thermal effect induced by a lower Q factor. For the same reason, the threshold power may also increase. Both the decrease of red-shift and increase of the threshold power are unfavorable to the performance of optical power limiter. Thus there is a trade-off between the operation bandwidth and the power limit performance in the choice of Q factor.

In addition, we noted that two photon absorption (TPA) and free carrier absorption (FCA) may act as important roles in silicon micro-cavity, as Johnson et al. mentioned in their previous work^28^. But these significant effects only occur when the Q factor is very high, and mode volume and thermal resistance is very small. In our case, despite of the high power injection, the MRR Q factor (~1.8*10^4^) is not high enough for TPA and FCA to induce blue-shift effect on the performance of the power limiter, compared to the micro-cavity structures in Ref. 28. Meanwhile, due to the 12 micron radius of the MRR, mode volume and thermal resistance of the MRR is also relatively large, which further reduce the effect of TPA and FCA. Thus the observed performance of the optical power limiter is almost free from TPA and FCA.

In summary, we experimentally demonstrate an all-passive chip-integrated optical power limiter which is capable of limiting the output power and protect vulnerable optical device from high power damage without deteriorating the transmitted signal. The device can be easily fabricated and completely compatible with CMOS technology. Additionally, we measure the device's performance under 10 Gbit/s and 20 Gbit/s and analyze the relationship of the device performance and the signal bit rates. We also figure out that the device's operation bandwidth is limited by MRR's 3 dB bandwidth. We believe this all-passive chip-scale device can play an indispensable role in the future optical network and enable more complicated, flexible, large-scale and intelligent photonic integration system.

## Methods

Devices fabrication. We employ a single add-drop MRR as the all-passive optical power limiter. We design and fabricate the MRR on an SOI wafer. The top silicon thickness of SOI wafer is 340 nm and the buried oxide layer thickness is 2 μm. The device layout was transferred to ZEP520A photoresist by E-beam lithography. Then the upper silicon layer is etched downward for 240 nm to form silicon ridge waveguide and input/output grating couplers, through inductively coupled plasma (ICP) etching (Oxford Instruments Plasmalab System 100). We use vertical grating coupling method to couple the fiber and the silicon MRR. The coupling loss of the grating coupler was measured to be 5 dB for single side. The whole fabrication process is done using CMOS compatible processes.

The red-shift measurement: The CW wavelength is aligned to the MRR resonant wavelength, i.e., 1557.87 nm. Due to the existence of ASE source, red shift can be measured conveniently by analyzing the spectrum around next resonant wavelength, i.e., 1548.3 nm. In this way, we can measure both signal power and red shift of MRR spectrum accurately.

## Author Contributions

J.J.D., S.Q.Y. proposed the study. A.L.Z. and X.L.Z. fabricated the device. S.Q.Y. and A.L.Z. carried out the experiment. S.Q.Y. analyzed the results and wrote the manuscript. J.J.D. supervised the project and edited the manuscript. All authors discussed the results and commented on the manuscript.

## Figures and Tables

**Figure 1:**
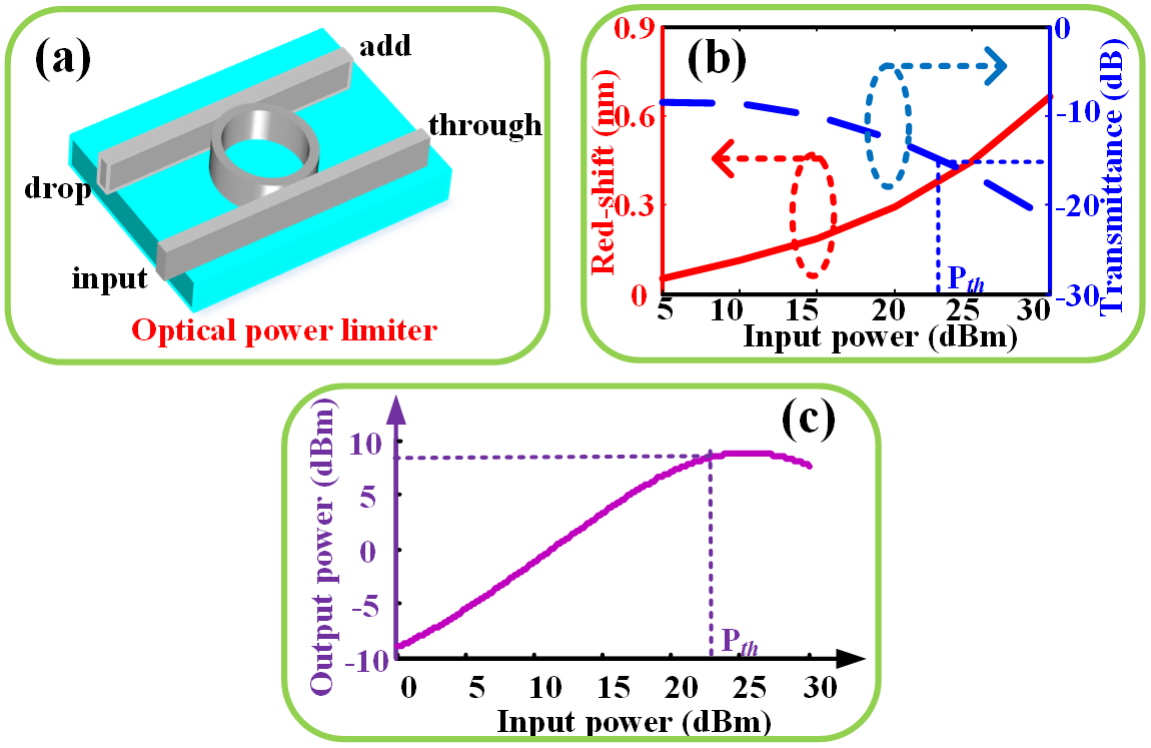
General view and calculations of optical power limiter. (a) General view of optical power limiter, (b) red curve: calculated red-shift as a function of input power, blue curve: transmittance at drop port as function of input power, (c) calculated output power as a function of input power.
